# Transcriptomic and Metabolomic Profiling of Root Tissue in Drought-Tolerant and Drought-Susceptible Wheat Genotypes in Response to Water Stress

**DOI:** 10.3390/ijms251910430

**Published:** 2024-09-27

**Authors:** Ling Hu, Xuemei Lv, Yunxiu Zhang, Wanying Du, Shoujin Fan, Lingan Kong

**Affiliations:** 1 Institute of Environment and Ecology, Shandong Normal University, Jinan 250014, China; hu_ling_123@163.com; 2 Crop Research Institute, Shandong Academy of Agricultural Sciences, Jinan 250100, China; 3 College of Life Sciences, Shandong Normal University, Jinan 250014, China

**Keywords:** *Triticum aestivum*, molecular mechanism, osmotic adjustment, drought tolerance

## Abstract

Wheat is the most widely grown crop in the world; its production is severely disrupted by increasing water deficit. Plant roots play a crucial role in the uptake of water and perception and transduction of water deficit signals. In the past decade, the mechanisms of drought tolerance have been frequently reported; however, the transcriptome and metabolome regulatory network of root responses to water stress has not been fully understood in wheat. In this study, the global transcriptomic and metabolomics profiles were employed to investigate the mechanisms of roots responding to water stresses using the drought-tolerant (DT) and drought-susceptible (DS) wheat genotypes. The results showed that compared with the control group, wheat roots exposed to polyethylene glycol (PEG) had 25941 differentially expressed genes (DEGs) and more upregulated genes were found in DT (8610) than DS (7141). Gene Ontology (GO) and Kyoto Encyclopedia of Genes and Genomes (KEGG) analysis showed that the DEGs of the drought-tolerant genotype were preferably enriched in the flavonoid biosynthetic process, anthocyanin biosynthesis and suberin biosynthesis. The integrated analysis of the transcriptome and metabolome showed that in DT, the KEGG pathways, including flavonoid biosynthesis and arginine and proline metabolism, were shared by differentially accumulated metabolites (DAMs) and DEGs at 6 h after treatment (HAT) and pathways including alanine, aspartate, glutamate metabolism and carbon metabolism were shared at 48 HAT, while in DS, the KEGG pathways shared by DAMs and DEGs only included arginine and proline metabolism at 6 HAT and the biosynthesis of amino acids at 48 HAT. Our results suggest that the drought-tolerant genotype may relieve the drought stress by producing more ROS scavengers, osmoprotectants, energy and larger roots. Interestingly, hormone signaling plays an important role in promoting the development of larger roots and a higher capability to absorb and transport water in drought-tolerant genotypes.

## 1. Introduction

Climate change has caused severe drought, which is the primary environmental factor restricting sustainable agriculture, resulting in significant reductions in food productivity in the world [1]. Under water deficit, turgor pressure and osmotic potential changes are perceived by plants and serve as signals participating in the biophysical and molecular response process, leading to the activation of drought-responsive genes in the roots [2].

Plants first recognize water deficit by roots and then transmit several molecular signals from roots to shoots [2]. Modification of root configuration is an essential strategy for plants to cope with abiotic stresses [3]. For instance, drought stress induces premature differentiation of the root apical meristem, resulting in a reduction in primary root length and an increase in lateral root number in wheat (*Triticum aestivum* L.) [4]. The root system with an increased surface area facilitates water and nutrient uptake in plants and thereby enables their survival under water deficit [5].

ROS levels can rise significantly when plants are subjected to various abiotic stresses including drought, heat, salt and chilling stresses, resulting in oxidative damage to protein, DNA and lipids and thereby affecting normal cellular functions, which may eventually cause cell death [6]. Malondialdehyde (MDA), as a biomarker of lipid peroxidation in the plasma membrane, was commonly used to evaluate plant tolerance to various stresses [7]. An antioxidant system, comprising enzymatic and non-enzymatic components, readily scavenges the ROS and thereby protects plants from oxidative damage [8]. Superoxide dismutase (SOD), catalase (CAT), peroxidase (POD), ascorbate peroxidase (APX), glutathione peroxidases (GPX) and glutathione-S-transferase (GST) were involved in enzymatic antioxidant systems. Among them, SOD constitutes the first line of defense against ROS damage. It converts the superoxide anion radical (O_2_^•−^) into O_2_ and hydrogen peroxide (H_2_O_2_); the latter is then converted into harmless product water by action of CAT and POD [9,10,11]. Non-enzymatic metabolites, such as flavonoids and anthocyanins, also play an important role in protecting plant cells against ROS damage caused by radiation [12] and drought [13].

Under a water deficit, the deposition of lignin in the cell wall enables plants to long-distance transport of water by enhancing mechanical support, which is crucial for plant growth [14]. The accumulation of lignin through either transcriptional or enzymatic modifications can confer tolerance to dehydration stress in rice (*Oryza sativa*) and Arabidopsis [15,16,17]. During root development, suberin deposition along the endodermis acts as a physical barrier, which restricts the efflux of water and nutrients through complex hormonal and transcriptional networks [18]. The development of the suberin lamellar layer formed through the deposition of suberin improved the drought tolerance of rice [19], wheat [20] and Arabidopsis [21,22] during osmotic stress.

Osmoprotectants enable the plant to regulate cellular osmotic adjustment, prevent membrane injury and protect protein and enzymes from damage caused by ROS [23]. Under water deficit, high levels of soluble sugars, such as sucrose and trehalose, can reduce the adverse effects of drought on root or leaf growth in the drought-tolerant genotypes of Arabidopsis [24,25], maize [26,27] and rice [28,29]. Proline, the most important osmolyte and signaling molecule, generally accumulates in the cytosol and improves protein stability by binding to hydrogen bonds without affecting the other functions under drought [30,31], salt [32], metal [33,34] and heat stress [35] in wheat.

Wheat, one of the widely grown staple crops, occupies over 220 million hectares of arable land, produces approximately 750 million tons per year and accounts for more than 20% of global daily protein and calorie intake [36]. However, the water deficit may lead to a dramatic reduction in wheat production [37,38]. Transcriptomic analysis, a widely used approach to investigate the relationship between genes and functions, has been commonly used to reveal mechanisms for drought-induced plant response in the roots of wheat [39,40], rice [41,42,43], barley [44], alfalfa [45], peanut [46] and chickpea [47]. The metabolites, the ultimate products of gene transcription and protein translation, form a bridge between genes and phenotypes and directly reflect the physiological responses in plants [48]. Metabolomic analyses have been frequently used to identify key metabolites in rice [49], maize [50], alfalfa [27], chickpea [51] and *Triticeae* crops under water deficit [31,52]. However, the regulatory mechanisms of plants’ responses to stresses are extremely complex and therefore, transcriptomic or metabolomic analysis alone cannot comprehensively understand the underlying mechanisms of plants’ responses to abiotic stresses.

In recent years, several studies have used integrated transcriptomics and metabolomics analyses to clarify the mechanisms under salt [53], heat [54] and drought [55] stresses in plants. In wheat, one study, based on the integrated transcriptomic and metabolomic approaches, suggests that increased antioxidant activity and flavonoid contents in leaf tissue lead to enhanced amino acid biosynthesis and ROS scavenging under water deficit [56]. Another study also used this method and uncovered the prominent pathways, such as plant hormone signaling, ABC transporter and arginine and proline metabolism in wheat leaves in response to drought stress [57]. Although some specific genes and key metabolites had been identified in leaves, the construction network for water-deficit resistance using transcriptomics and metabolomics had not been fully understood in wheat roots.

The wheat JM262 cultivar shows strong drought resistance at both the physiological and molecular levels [58], while the YN24 cultivar is ultrasensitive to drought. In this study, we investigated the dynamic metabolomic and transcriptomic profiles of wheat root tissues, aiming to identify the corresponding differentially expressed genes and potential metabolites at the molecular and metabolic levels to provide a valuable foundation for uncovering the regulatory mechanisms using the PEG drought-simulation method.

## 2. Results

### 2.1. Dry Weight and Water Contents

**Figure 1:**
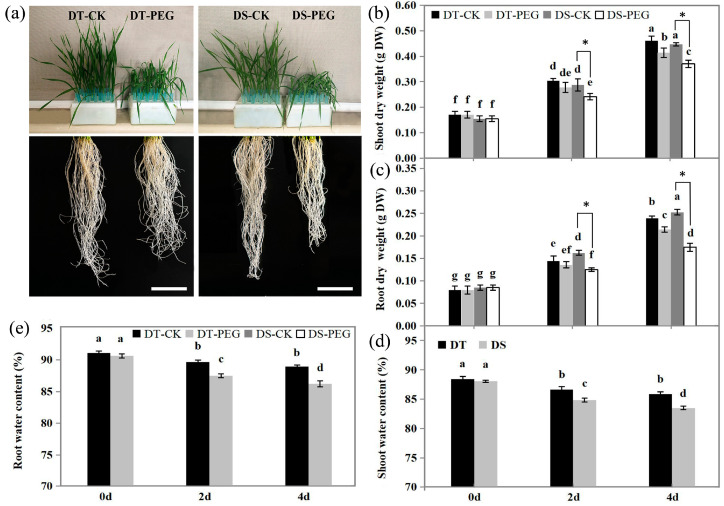
Effects of drought stress on the shoots and roots phenotype characterization (**a**) at 4 DAT, dry weight (scale bar of root is 5 cm), (**b**,**c**) and water contents (**d**,**e**) at 0, 2 and 4 DAT in DT and DS. Values are means ± SD from three biological replicates each with 10 plants (**a**) or with six plants (**b**–**e**). According to Tukey’s significant deference test using SPSS software, bars with the different letters are significantly different at *p* < 0.05 and bars marked with an asterisk (*) are significantly different at *p* < 0.01. DT-CK: JM262 grown in control conditions; DT-PEG: JM262 treated with PEG; DS-CK: YN24 grown in control conditions; DS-PEG: YN24 treated with PEG.

Before PEG treatment, the dry weights of shoots and roots of wheat seedlings grown in Hoogland’s nutrient solution showed no differences between DT and DS. At 4 days after treatment (DAT), the dry weights of shoots and roots were significantly higher in DT than DS (*p* < 0.05) (Figure 1a–c). Compared with controls, dry weights of shoots and roots were significantly decreased in DS at 2 and 4 DAT (*p* < 0.01) and only at 4 DAT (*p* < 0.05) in DT. At 4 DAT, the dry weights in DT were significantly decreased by 10.87% (*p* < 0.05) and 12.50% (*p* < 0.05) in shoots and roots, respectively and by 17.78% (*p* < 0.01) as well as by 32.02% (*p* < 0.01) in DS compared with their respective controls.

The water contents of shoots and roots showed no difference between DT and DS before PEG treatment while being significantly higher in DT than DS throughout the PEG treatment (*p* < 0.05) (Figure 1d,e). At 2 DAT, the water contents of shoots and roots in both genotypes were sharply decreased in comparison to 0 DAT (*p* < 0.05), with 2.02% (*p* < 0.05) and 1.56% (*p* < 0.05) decreases in DT and 3.61% (*p* < 0.05) and 3.44% (*p* < 0.05) decreases in DS in shoots and roots, respectively, compared with their respective controls. At 4 DAT, the shoot and root water contents in DS continuously and significantly decreased compared with 2 DAT (*p* < 0.05), while no significant change was observed in DT.

### 2.2. Root System Architecture

**Figure 2:**
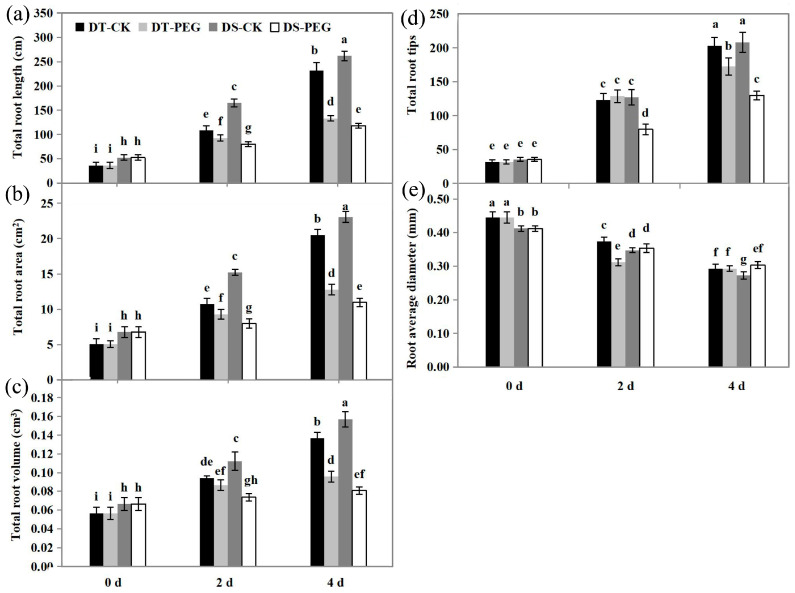
Effects of drought stress on root structure in DT (JM262) and DS (YN24), such as the total root length (**a**), total root area (**b**), total root volume (**c**), total root tips (**d**) and root average diameter (**e**) at 0, 2 and 4 DAT. Values are means ± SD from six biological replicates each with one plants. Bars with different letters are significantly different (*p* < 0.05) according to Tukey’s significant deference test using SPSS software. DT-CK: JM262 grown in control conditions; DT-PEG: JM262 treated with PEG; DS-CK: YN24 grown in control conditions; DS-PEG: YN24 treated with PEG.

Before PEG treatment, the values of total root length (RL), root volume (RV), root surface area (RA) and number of root tips (RT) were lower in DT than DS (*p* < 0.05), while being significantly higher in DT than in DS at 2 and 4 DAT (*p* < 0.05) (Figure 2a–c). Compared to control conditions, the values of RL, RA, RV and RT were much lower at 2 and 4 DAT (*p* < 0.05) in both genotypes after PEG treatment, except for the RT in DT, which showed no difference at 2 DAT (Figure 2d). The value of root average diameter (RAD) was higher in DT than DS before PEG treatment, while it was significantly lower in DT than DS at 2 DAT. Compared to 0 DAT, the RAD of both genotypes gradually decreased at 2 and 4 DAT (*p* < 0.05) (Figure 2e).

### 2.3. The Concentrations of O_2_^•−^, H_2_O_2_ and MDA

**Figure 3:**
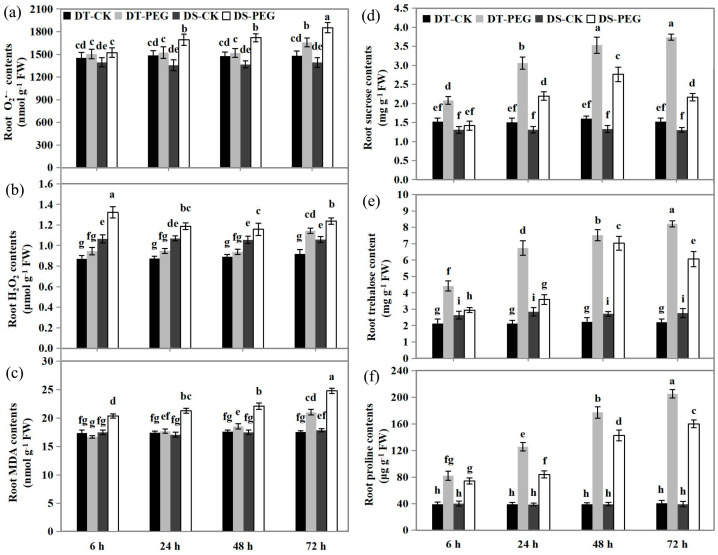
Effects of PEG treatment on the contents of root O_2_^•−^ (**a**), H_2_O_2_ (**b**), MDA (**c**), sucrose (**d**), trehalose (**e**) and proline (**f**) in DT (JM262) and DS (YN24) at 6, 24, 48 and 72 HAT. Values are means ± SD from three biological replicates, each with 0.5 g samples. Bars with different letters are significantly different at *p* < 0.05 according to Tukey’s significant deference test using SPSS software. DT-CK: JM262 grown in control conditions; DT-PEG: JM262 treated with PEG; DS-CK: YN24 grown in control conditions; DS-PEG: YN24 treated with PEG.

Before PEG treatment, the concentration of O_2_^•−^ was slightly higher in DT than in DS, while it was significantly (*p* < 0.05) lower in DT than in DS especially from 24 to 72 HAT. After PEG treatment, the concentration of O_2_^•−^ in DT showed no significant changes from 6 to 48 HAT, while in DS it significantly (*p* < 0.05) increased from 6 to 72 HAT (Figure 3a). The H_2_O_2_ concentration in DT maintained control levels from 6 to 48 HAT and then increased significantly (*p* < 0.05) at 72 HAT, while in DS, the H_2_O_2_ concentration increased significantly (*p* < 0.05) during the PEG treatment (Figure 3b). Compared to DS, DT showed a lower H_2_O_2_ concentration under both PEG and control conditions (*p* < 0.05). As a consequence, the MDA level showed a similar trend with O_2_^•−^ in both genotypes after PEG treatment (Figure 3c).

### 2.4. The Concentrations of Sucrose, Trehalose and Proline

Before PEG treatment, the sucrose concentrations showed no significant difference between the two genotypes, while trehalose concentrations of DT were significantly (*p* < 0.05) lower than DS (Figure 3d,e). After PEG treatment, the concentration of both sucrose and trehalose increased gradually from 6 HAT to 72 HAT in DT (*p* < 0.05), whereas it increased from 6 HAT to 48 HAT and then decreased at 72 HAT in DS. In addition, DT showed consistently higher sucrose and trehalose concentrations than DS under PEG treatment (*p* < 0.05) (Figure 3d,e). The root proline concentrations were gradually increased in both genotypes from 6 HAT to 72 HAT, but DT showed consistently much higher levels than DS (Figure 3f).

### 2.5. Comparison of Transcriptional Profiling between DT and DS in Response to PEG Treatment

**Figure 4:**
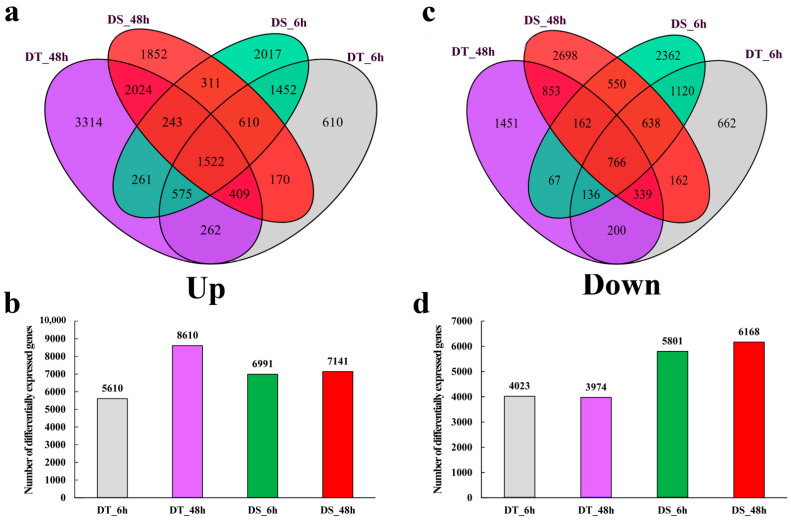
Venn diagram of DEGs between DT (JM262) and DS (YN24). (**a**,**b**): Upregulated genes at 6 and 48 HAT. (**c**,**d**): Downregulated genes at 6 and 48 HAT. Three biological replicates (each with 10 plants) were performed for the two genotypes.

A set of 24 samples yielded more than 1.7 billion clean reads. The high-throughput sequencing data reveals that over 92% of the reads achieved a Q30 level, indicating an error probability of 0.1% (Table S1). The mapping rate from each sample is shown in Table S2. To investigate drought-responsive genes in wheat, genes with an FDR < 0.05 and a |log_2_FC (fold changes)| ≥ 1 were defined as DEGs. In total, 25,941 DEGs were identified by a method of pair-wise comparison between normal and PEG conditions in each sample at 6 and 48 HAT (Table S3). As shown in Figure 4, the number of upregulated DEGs was much more in DT (8610) than in DS (7141), especially at 48 HAT (Figure 4a,b), while DS showed much more downregulated DEGs (11,969) than in DT (7997) during the drought treatment (Figure 4c,d).

### 2.6. GO and KEGG Enrichment of DEGs

**Figure 5:**
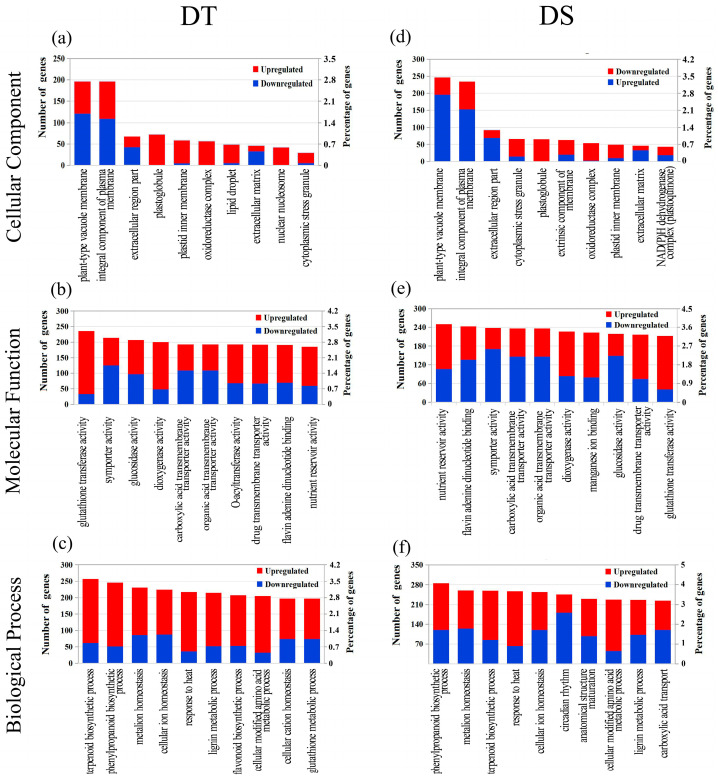
The top 10 GO terms for categories of biological process, cellular component and molecular function in DT (**a**–**c**) and DS (**d**–**f**). The left y-axis represents the number of enriched DEGs and the right y-axis is the percentage (%) of DEGs. The red and blue bar plots represent the number of upregulated and downregulated DEGs, respectively.

To understand the biological function of the drought-induced DEGs, GO enrichment was performed using an FDR-adjusted *p*-value < 0.05 as the cut-off. All the DEGs were divided into the biological process, cellular component and molecular function categories, the Top 10 GOs in DT and DS are shown in Figure 5. For cellular component analysis, the most enriched GO terms were plant-type vacuole membrane (GO:0009705) and integral component of the plasma membrane (GO:0005887) in both genotypes (Figure 5a,d). Interestingly, these two GO terms included approximately 50% DEGs in the top 10 GO terms in both genotypes. In addition, eight GO terms were found to overlap in both genotypes, while two terms such as lipid droplet (GO:0005811) and nuclear nucleosome (GO:0000788) were specifically annotated in DT. Additionally, the overlapped GO terms showed almost more upregulated and less downregulated DEGs in DT than in DS. In the molecular function category, the top three GO terms were glutathione transferase activity (GO:0004364), symporter activity (GO:0015293) and glucosidase activity (GO:0015926) in DT, while in DS, they were nutrient reservoir activity (GO:0045735), flavin adenine dinucleotide binding (GO:0050660) and symporter activity (GO:0015293) (Figure 5b,e). The overlapped GO terms showed more upregulated or less downregulated DEGs in DT than DS. In the biological process category, seven overlapped GO categories showed a significantly higher number of upregulated and a lower number of downregulated DEGs in DT compared to DS (Figure 5c,f). Among these overlapped GO categories, the terpenoid biosynthetic process (GO:0016114), phenylpropanoid biosynthetic process (GO:0009699) and metal ion homeostasis (GO:0055065) were the top three GO terms in both genotypes. In addition, the flavonoid biosynthetic process (GO:0009813), cellular cation homeostasis (GO:0030003) and glutathione metabolic process (GO:0006749) were specifically annotated in DT.

**Figure 6:**
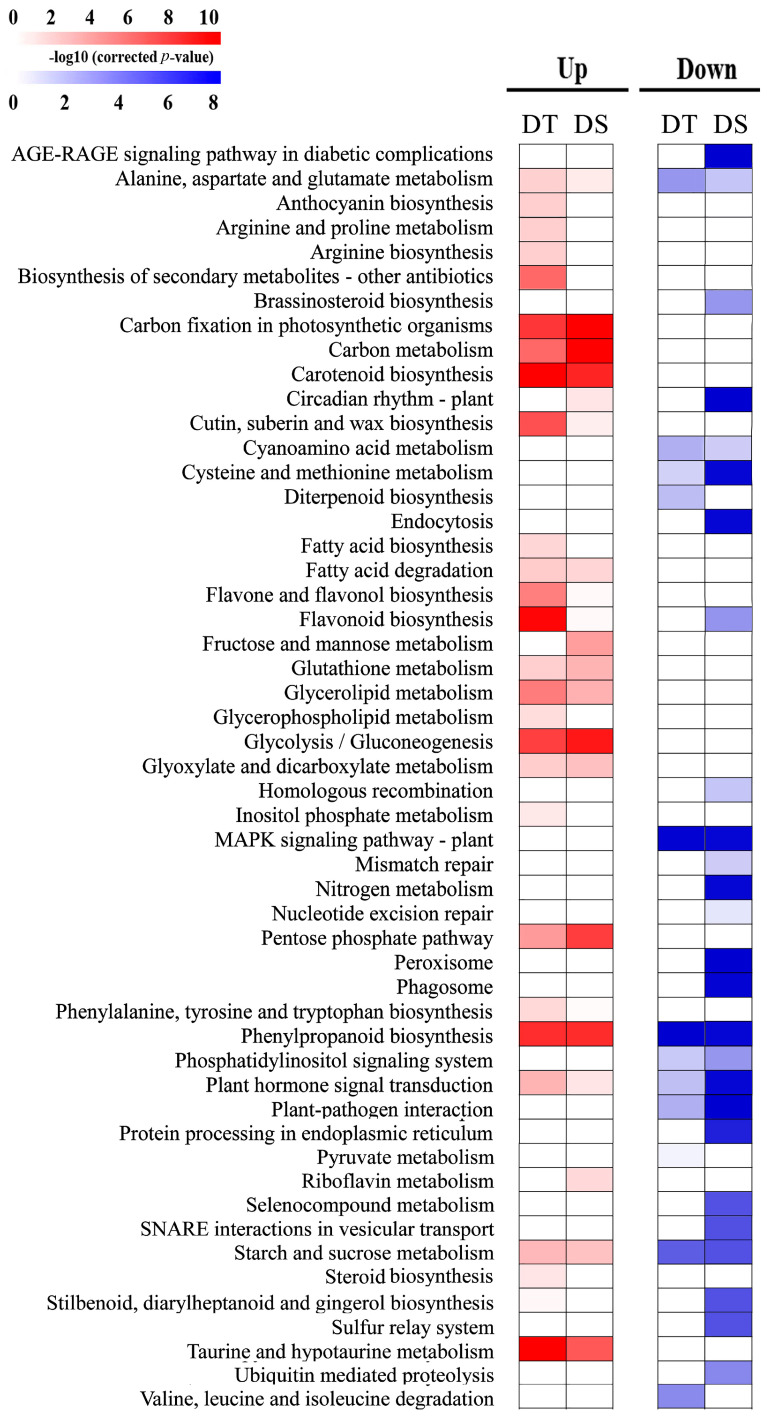
KEGG pathway affected by drought stress in DT (JM262) and DS (YN24). KEGG pathway enrichment analysis was performed for lists of significantly up- and down-regulated genes for each genotype. The heatmap presents statistical significance by log_10_(corrected *p*-value).

KEGG analysis was used to identify potential biological pathways represented in the wheat root transcriptome. There were 9478 DEGs assigned to 52 pathways (Figure 6) and 11 pathways were observed as preferably enriched in upregulated DEGs in DT, and three of 11 were most enriched, including flavone and flavonol biosynthesis, flavonoid biosynthesis and biosynthesis of secondary metabolites-other antibiotic. A total of 15 pathways were upregulated in both genotypes, among which the cutin, suberin and wax biosynthesis pathways exhibited significantly higher enrichment in DT than DS after PEG treatment. The number of the pathways enriched from downregulated DEGs was higher in DS (27) than DT (11) and 3 of 9 overlapped downregulated pathways including cysteine and methionine metabolism, plant hormone signal transduction and plant-pathogen interaction were much more highly enriched in DS than DT (Figure 6).

### 2.7. Enzymatic Antioxidants

**Figure 7:**
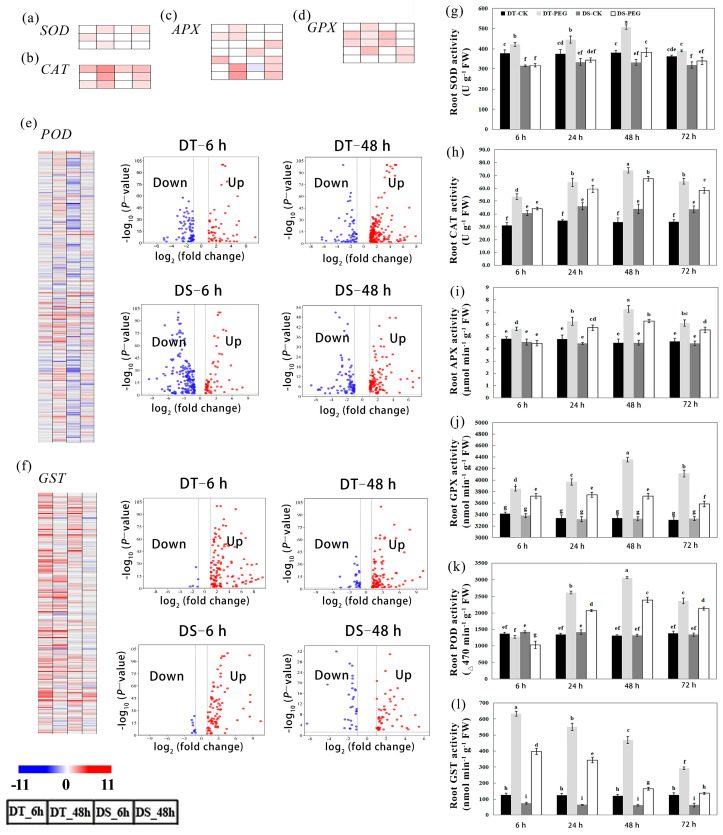
Effects of drought stress on the root antioxidant activities. Heatmap of DEGs related to *SOD* (**a**), *CAT* (**b**), *APX* (**c**) and *GPX* (**d**). Volcanic and heatmap of DEGs related *POD* (**e**) and *GST* (**f**). The activities of SOD (**g**), CAT (**h**), APX (**i**), GPX (**j**), POD (**k**) and GST (**l**). Relative levels of expression are shown by a color gradient from low (blue) to high (red) in genes. The squares are ordered from left to right: DT_6 h, DT_48 h, DS_6 h and DS_48 h. The numbers in the scale bar stand for the log_2_(FC) in expression. Values are means ± SD from three biological replicates, each with 0.5 g samples. Bars with different letters are significantly different at *p* < 0.05 according to Tukey’s significant deference test using SPSS software. DT-CK: JM262 grown in control conditions; DT-PEG: JM262 treated with PEG; DS-CK: YN24 grown in control conditions; DS-PEG: YN24 treated with PEG.

The transcriptomic analysis showed that expression of DEGs including *SOD*, *CAT*, *POD*, *APX* and *GST* were activated in response to water stress and that almost all the DEGs such as *SOD*, *CAT*, *APX* and *GPX* were upregulated in both genotypes at 6 and 48 HAT. In addition, three, five, six and six genes including *SOD*, *CAT*, *APX* and *GPX*, respectively, in DT and one, four, five and four of these genes in DS were upregulated (Figure 7a–d; Table S4). A total of 235 and 162 *POD* genes were upregulated in DT and DS, respectively, while 133 and 245 were downregulated after PEG treatment (Figure 7e; Table S4). Additionally, the number of upregulated *POD* genes was increased, while the number of downregulated genes was decreased in both genotypes at 48 HAT compared to 6 HAT. The expression of a total of 232 *GST* genes were upregulated in DT compared to 157 in DS after PEG treatment, while 28 genes were downregulated in DT compared to 34 in DS (Figure 7f, Table S4). In addition, compared to 6 HAT in both genotypes, the number of upregulated genes decreased at 48 HAT, while the number of downregulated genes increased.

The physiological assay showed that the activity of root SOD, CAT, APX, and GPX was significantly (*p* < 0.05) higher in DT than in DS after PEG treatment. Interestingly, we found the activity of SOD, CAT and APX showed the same trend in both genotypes during PEG treatment, namely, the activity increased with increasing treatment time, peaked at 48 HAT and then decreased at 72 HAT (*p* < 0.05) (Figure 7g–j). The POD activity was decreased slightly in DT while it decreased significantly in DS (*p* < 0.05) compared with their respective control conditions at 6 HAT. In addition, the POD activity was significantly higher in DT than in DS throughout the PEG treatment (*p* < 0.05) (Figure 7k). Interestingly, POD activity showed the same trend in both genotypes, namely, the activity gradually increased with increasing time and peaked at 48 HAT; it decreased at 72 HAT. After PEG treatment, the GST activity was significantly higher in DT than DS (*p* < 0.05) (Figure 7l). The maximum level of root GST activity of both genotypes was observed at 6 HAT and then decreased gradually from 6 to 72 HAT.

### 2.8. Biosynthesis of Plant Hormones

**Figure 8:**
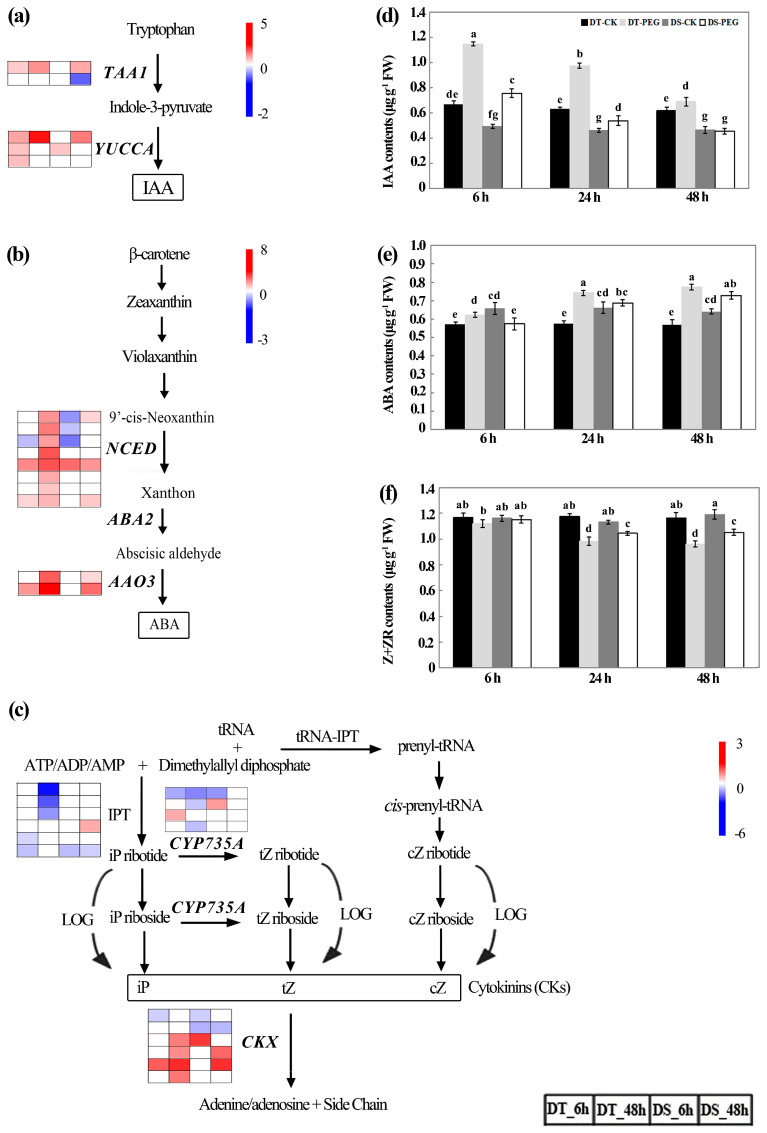
DEGs involved in the biosynthesis of IAA (**a**), ABA (**b**) and CKs (**c**) during PEG treatment and contents of root IAA (**d**), ABA (**e**) and Z + ZR (**f**). Relative levels of expression are shown by a color gradient from low (blue) to high (red) in genes. The squares are ordered from left to right: DT_6 h, DT_48 h, DS_6 h and DS_48 h. The numbers in the scale bar stand for the log_2_(FC) in expression. Values are means ± SD from three biological replicates, each with 0.5 g samples. Bars with different letters are significantly different at *p* < 0.05 according to Tukey’s significant deference test using SPSS software. DT-CK: JM262 grown in control conditions; DT-PEG: JM262 treated with PEG; DS-CK: YN24 grown in control conditions; DS-PEG: YN24 treated with PEG.

A total of five DEGs that were involved in IAA biosynthesis were identified after PEG treatment. Two genes were annotated to be L-tryptophan-pyruvate aminotransferase 1 (*TAA1*), among which, one was upregulated at both 6 and 48 HAT in DT, while only one was upregulated at 48 HAT in DS; another one was not affected in DT at both 6 and 48 HAT, while it was downregulated in DS at 48 HAT (Figure 8a; Table S5). Three indole-3-pyruvate monooxygenase (*YUCCA*) genes showed upregulated in DT at 6 HAT, while only one gene was upregulated in DS. At 48 HAT, one *YUCCA* gene was upregulated in both genotypes and DT showed higher expression levels than DS. Physiological determination showed that the root IAA contents were significantly higher in DT than in DS under both control and PEG treatment conditions (*p* < 0.05). In both genotypes, root IAA contents were significantly increased at 6 HAT and then gradually decreased from 24 to 48 HAT compared with the control. In particular, it decreased to the control level in DS at 48 HAT (*p* < 0.05). Nevertheless, IAA contents still showed higher levels of DT under PEG treatment compared with control conditions (*p* < 0.05). (Figure 8d).

9-cis-epoxycarotenoid dioxygenase (NCED) and abscisic-aldehyde oxidase (AAO) were key enzymes in ABA biosynthesis [59]. Our RNA-seq analysis revealed that two and eight genes annotated to be *NCED* were upregulated in DT roots at 6 and 48 HAT, respectively, while one and three genes were upregulated in DS (Figure 8b; Table S5). The *NCED* genes were downregulated only at 6 HAT in both genotypes; however, only one of these genes was downregulated in DT compared to three in DS. After PEG treatment, two genes annotated to be *AAO3* were upregulated in both genotypes. Moreover, the expression levels of overlapped upregulated genes showed much higher levels in DT than DS, in particular at 48 HAT. The root ABA contents were significantly lower in DT than in DS (*p* < 0.05) before PEG treatment; however, it was significantly and consistently higher in DT than in DS after PEG treatment. The ABA contents significantly increased at 6, 24 and 48 HAT in DT, while it significantly increased at 48 HAT in DS (*p* < 0.05) (Figure 8e).

Adenylate dimethylallyltransferase (*IPT*) genes and cytokinin trans-hydroxylase (*CYP735A*) genes were involved in cytokinin (CK) synthesis, while cytokinin dehydrogenase (*CKX*) genes were involved in CK degradation. In our study, the number of downregulated *IPT* genes was much more in DT (5) than in DS (2) at 6 and 48 HAT (Figure 8c; Table S5). A total of four *CYP735A* genes were downregulated in DT compared to one in DS and one *CYP735A* gene was upregulated in both genotypes at 6 HAT. A total of five *CKX* genes were upregulated in DT compared to three in DS and one gene was downregulated in DT compared to three in DS. Interestingly, in both genotypes, a greater number of *CKX* genes were upregulated and a smaller number of genes were downregulated at 48 HAT compared to 6 HAT. As a consequence, the root Z + ZR contents were decreased significantly at 24 and 48 HAT (*p* < 0.05), while no significant difference was observed at 6 HAT in both genotypes compared with their controls and the root Z + ZR contents were consistently lower in DT than DS at 24 and 48 HAT (Figure 8f).

### 2.9. DEGs Related to Plasma Membrane Aquaporins (AQPs)

*PIP1s* genes were totally downregulated in both genotypes. Seven and three *PIP2s* genes were upregulated in DT and DS, respectively, while two and four genes were downregulated. In addition, one *OsPIP2;5* gene and three *OsPIP2;7* genes were upregulated at 48 HAT in both genotypes; however, the expression levels of these genes were much higher in DT than in DS. In addition, six *TIPs* genes were upregulated at 6 and one at 48 HAT and three *TIPs* genes were downregulated at each time point in DT. In DS, *TIPs* genes were all downregulated throughout PEG treatment (Table S6).

To validate the RNA-seq data, the gene expression changes in 12 drought-related DEGs were analyzed by qRT-PCR (Table S7). Although the fold changes in selected genes were different between RNA-seq and qRT-PCR, the changes in qRT-PCR were consistent with the results of RNA-seq (Figure S1).

### 2.10. DEGs Associated with the Suberin Biosynthesis

**Figure 9:**
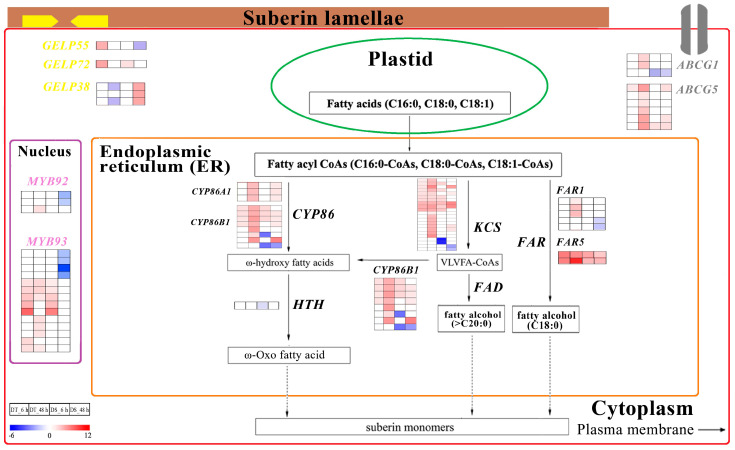
The pathway of suberin biosynthesis. Genes were displayed in different colors. Relative levels of expression are shown by a color gradient from low (blue) to high (red). The squares are ordered from left to right: DT_6 h, DT_48 h, DS_6 h and DS_48 h. The numbers in the scale bar stand for the log_2_FC(fold changes) in expression.

In KEGG analysis, the suberin biosynthesis pathway showed much more enriched in DT than in DS (Figure 7). In this pathway, a total of 61 and 46 DEGs were identified in DT and DS throughout PEG treatment, including β-Ketoacyl-CoA synthase (*KCS*) genes, fatty acyl-CoA reductase (*FAR*) genes and fatty acid omega-hydroxylase (*CYP86*) genes (Figure 9; Table S8). In addition, the ATP-binding cassette (ABC) G family (*ABCG*) genes and GDSL-type esterase/lipase proteins (*GELP*) genes were also deferentially expressed in both genotypes under PEG treatment. A greater number of *KCS* were upregulated in DT (28) than in DS (13). The expression of a total of three DEGs *CYP86A1* was upregulated in both genotypes and DT showed higher expression levels than DS. Of eight *CYP86B1* genes, six were upregulated in both genotypes and showed higher expression levels in DT than in DS, especially at 48 HAT. The remaining two *CYP86B1* genes were upregulated or not significantly changed in DT, while they were downregulated in DS. After PEG treatment, three *FAR1* genes were specially upregulated in DT and two genes were specially downregulated in DS. Two *FAR5* genes were all upregulated in the two genotypes, while the expression levels were higher in DT than in DS. The *ABCG1* genes were upregulated in DT, with no significant changes and even downregulated in DS. During PEG treatment, *ABCG5* genes were upregulated in both genotypes and the number of genes was greater in DT (7) than in DS (4). During PEG treatment, the genes *GELP55* and *GELP72* were both responsible for suberin homeostasis [60] and were upregulated in DT, whereas in DS, they showed a downregulated (*GELP55*) or lower upregulation level (*GELP72*) than in DT. The *GELP38* genes were downregulated especially at 48 HAT in DT, while all of the *GELP38* genes were upregulated in DS.

### 2.11. Metabolic Characteristics of Wheat Root Response to Drought Stress

**Figure 10:**
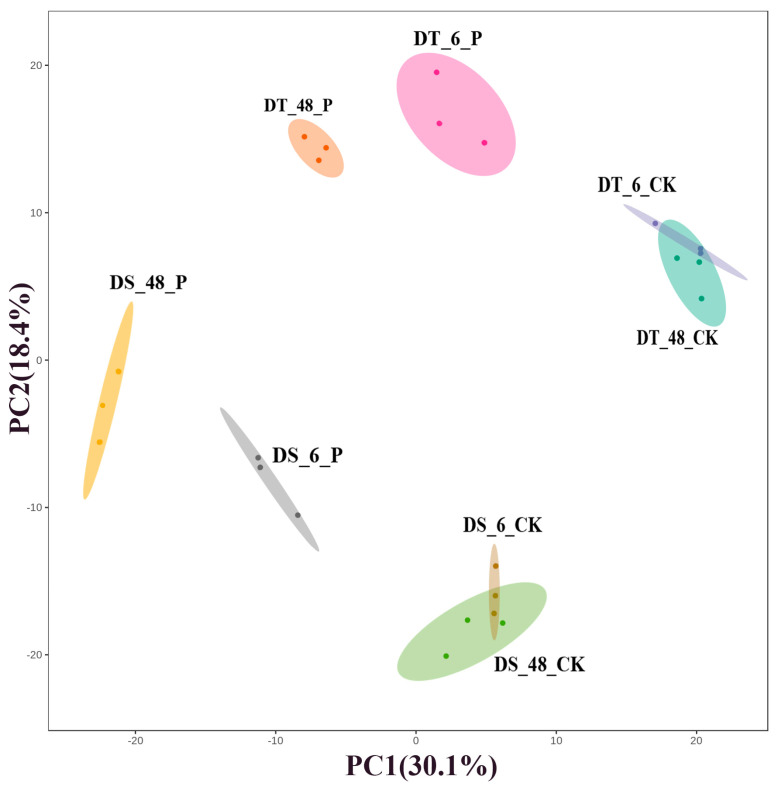
Principal component analysis of metabolic profiles of DT (JM262) and DS (YN24) under control and PEG treatment. Three biological replicates (each with 10 plants) were performed for the two genotypes.

To understand the overall metabolic differences between various groups, prior, we performed a principal component analysis (PCA) of the samples to screen for differential metabolites, reduce the dimensionality of the data and visualize the relationship among samples. The first principal component (PC1) explained 30.1% of the total variation, while the second principal component (PC2) explained 18.4% variation across the data set (Figure 10). The difference in metabolite levels between DT and DS was distinguished by the first principal component (PC1) and the second principal component (PC2) clearly separated control and PEG-treated samples.

A total of 271 DAMs were identified and there were 19 and 42 DAMs specifically found in response to drought for DT at 6 and 48 HAT, while 19 and 55 metabolites were unique in response to drought for DS at 6 and 48 HAT (Figure S2a, Table S9). The Venn diagram results revealed that 27 metabolites were produced in roots of both genotypes after PEG treatment, including five amino acids and their derivatives (1-methylhistidine, L-glutamic acid O-glucoside, aspartic acid di-O-glucoside, L-alanine and L-proline), two benzoic acid derivatives (2,5-dihydroxy benzoic acid O-hexside and anthranilate O-hexosyl-O-hexoside), three carbohydrates (D-(+)-sucrose and D(+)-Melezitose O-rhamnoside), three flavone C-glycosides (8-C-hexosyl-apigenin O-feruloylhexoside, Eriodictiol C-hexosyl-O-hexoside and C-hexosyl-chrysin O-feruloylhexoside), three flavonoids (tricin O-eudesmic acid, luteolin O-sinapoylhexoside and ayanin), two hydroxycinnamoyl derivatives (hydrocinnamic acid and caffeic aldehyde), one lipid glycerophospholipids (PC 16:1/14:1), four organic acids (5-hydroxyhexanoic acid, γ-aminobutyric acid, mandelic acid and Dl-2-aminooctanoic acid), two phenolamides (N-feruloyl spermidine and N-*p*-coumaroylspermine) and three quinate and its derivatives (O-*p*-coumaroyl quinic acid O-rutinoside derivative, 5-O-*p*-coumaroyl shikimic acid and 5-O-*p*-coumaroylquinic acid). In addition, there were 101 (60 up and 41 down) at 6 HAT, 141 (93 up and 48 down) at 48 HAT in DT, 95 (45 up and 50 down) at 6 HAT and 155 (91 up and 62 down) at 48 HAT in DS (Figure S2b, Table S9).

### 2.12. Integrated Transcriptome and Metabolome Analysis for DT and DS Responsive to Drought Stress

A coexpression network analysis of the transcriptome and metabolome was performed to further explore the relationship between DEGs and DAMs from the roots of both genotypes under PEG treatment. In this study, the DEGs and DAMs with PCC greater than 0.8 were selected. In DT, we used 85 DAMs and 2667 DEGs for association analysis at 6 HAT, while 110 DAMs and 3563 DEGs were used at 48 HAT. In DS, we used 88 DAMs and 3540 DEGs for association analysis at 6 HAT, while 157 DAMs and 4618 DEGs were used at 48 HAT (Table S10).

The KEGG pathways shared by DAMs and DEGs were examined (Table S9). In DT, a total of 64 and 107 pathways were enriched at 6 and 48 HAT, respectively, including 1585 DEGs and 36 DAMs at 6 HAT and 2141 DEGs and 58 DAMs at 48 HAT. The common pathways with *p*-value_Gene < 0.05 and *p*-value_Metabolite < 0.05 included flavonoid biosynthesis and arginine and proline metabolism at 6 HAT, alanine, aspartate, glutamate metabolism and carbon metabolism at 48 HAT. In DS, a total of 101 and 110 pathways were obtained at 6 and 48 HAT, respectively, including 2459 DEGs and 48 DAMs at 6 HAT, while 2120 DEGs and 70 DAMs at 48 HAT. The common pathways with *p*-value_Gene < 0.05 and *p*-value_Metabolite < 0.05 included arginine and proline metabolism at 6 HAT and biosynthesis of amino acids at 48 HAT (Table S10).

### 2.13. DEGs and DAMs Involved in the Biosynthesis of Phenylpropanoid, Flavonoids, Anthocyanin and Lignin

**Figure 11:**
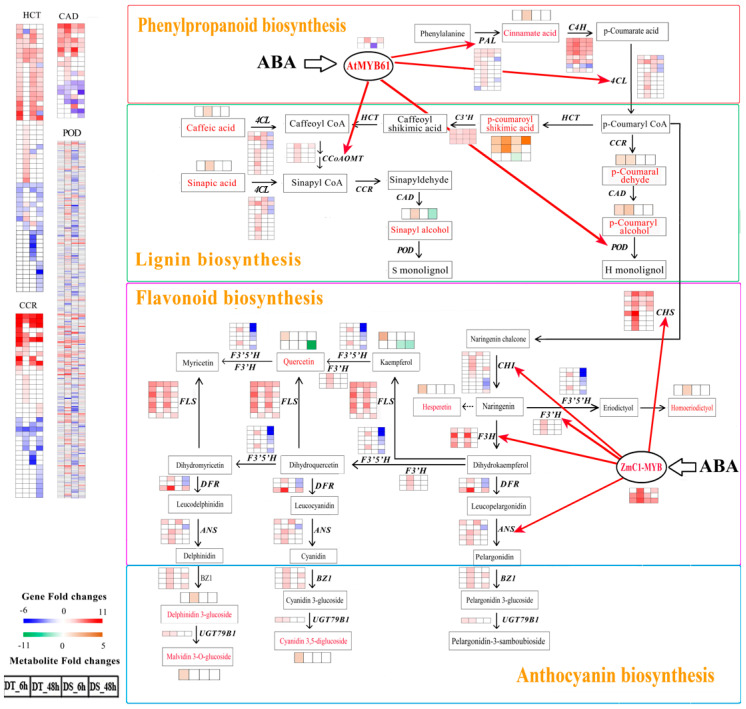
The pathways of phenylpropanoid biosynthesis, lignin biosynthesis, flavonoid biosynthesis and anthocyanin biosynthesis. Deferential expression of genes and accumulation of metabolites are presented by different colors. Relative levels of expression are shown by a color gradient from low (blue) to high (red) in genes and from low (green) to high (brown) in metabolites. The squares are ordered from left to right: DT_6 h, DT_48 h, DS_6 h and DS_48 h. The numbers in the scale bar stand for the log_2_FC(fold changes) in expression.

The flavonoid biosynthesis pathway was specially enriched at 6 HAT in DT by the correlation analysis of DEG and DAM (Table S10). The heat maps of expression for the DEGs and DAMs of this pathway in DT and DS at 6 and 48 HAT were drawn out (Figure 11). In the flavonoid biosynthesis pathway, 54 genes including chalcone synthase (*CHS*), chalcone isomerase (*CHI*), flavanone 3-hydroxylase (*F3H*), flavonoid 3′-hydroxylase (*F3′H*), flavonoid 3′,5′-hydroxylase (*F3′5′H*), flavonol synthase (*FLS*), dihydroflavonol 4-reductase (*DFR*) and anthocyanidin synthase (*ANS*) were differentially expressed in the roots of wheat under PEG treatment. In DT, all of the genes involved in the flavonoid biosynthetic pathway were upregulated, while three of these genes (*F3*′*5*′*H*, *ANS* and *DFR*) were downregulated in DS, especially at 48 HAT (Table S11). Additionally, the anthocyanin biosynthesis pathway was also specially enriched in DT according to the KEGG pathway. The *BZ1* genes were upregulated in both genotypes at 48 HAT and four DEGs were found in DT compared to two in DS. One *UGT79B1* gene was upregulated only in DT. The phenylpropanoid biosynthesis pathway, the upstream biosynthetic pathway for flavonoid biosynthesis and anthocyanin biosynthesis [61], showed significant enrichment in both genotypes according to the GO (Figure 5) and KEGG (Figure 6) analysis. The phenylalanine ammonia-lyase (*PAL*) genes, trans-cinnamate 4-monooxygenase (*C4H*) genes and 4-Hydroxycinnamoyl-CoA ligase (*4CL*) genes were almost upregulated in both genotypes at 6 HAT; at 48 HAT, these DEGs were still upregulated in DT, while in DS, the *PAL* and *4CL* genes were almost downregulated (Table S10). In the analysis of metabolic profiles, the cinnamate acid was specifically accumulated in DT after PEG treatment. In addition, the accumulation levels of DAMs including flavonols (kaempferol 3-O-galactoside and quercetin) and flavanones (homoeriodictyol and hesperetin) were especially increased in DT, while in DS, one kaempferol derivative (kaempferol 3-O-robinobioside) and one quercetin derivative (7-O-malonylhexosyl-hexoside) were especially downregulated. Three anthocyanin derivatives, including malvidin 3-O-glucoside, cyanidin 3,5-O-diglucoside and delphinidin 3-O-glucoside, were significantly accumulated only in DT (Figure 11, Table S9).

In the lignin biosynthesis pathway, the genes including cinnamoyl-CoA reductase (*CCR*), cinnamyl-alcohol dehydrogenase (*CAD*), shikimate O-hydroxycinnamoyltransferase (*HCT*), 3′-hydroxylase (*C3′H*), caffeoyl-CoA O-methyltransferase (*CCoAOMT*) and peroxidase (*POD*) showed differential expression in the roots of wheat under PEG treatment. In DT, 111 and 222 DEGs were upregulated at 6 and 48 HAT, respectively, compared to 104 and 139 DEGs in DS. At 6 and 48 HAT, 90 and 70 DEGs were, respectively, downregulated in DT compared to 160 and 116 DEGs in DS. In addition, four *CCoAOMT* genes were upregulated in DT while only one gene was upregulated in DS at 48 HAT. In the analysis of metabolic profiles, of three differentially accumulated shikimate derivatives under PEG treatment, two (5-O-*p*-coumaroyl shikimic acid, 3-O-*p*-coumaroyl shikimic acid) were accumulated in both genotypes and the accumulation levels were higher in DT than DS. The remaining derivative (5-O-*p*-coumaroyl shikimic acid O-hexoside) was downregulated only in DS. After PEG treatment, the accumulation levels of five DAMs classified as hydroxycinnamoyl derivatives (sinapic acid, sinapyl alcohol, *p*-coumaryl alcohol, *p*-coumaraldehyde and caffeic acid O-glucoside) were especially enriched in DT, while accumulation of these DAMs showed no significant changes and even downregulated in DS (Figure 11, Tables S8 and S11).

Except for these structure genes, the regulatory genes involved in phenylpropanoid and flavonoid biosynthesis, such as *ZmC1-myb* and *AtMyb61*, were differentially expressed in both DT and DS. Of the two *AtMyb61* genes, one was upregulated in both genotypes at 48 HAT and showed higher expression levels in DT than DS and another showed no significant changes in DT, while one was downregulated at 6 HAT in DS. The expressions of *ZmC1-myb* genes were upregulated in both genotypes and showed higher levels in DT than DS.

### 2.14. DEGs and DAMs Involved in Carbon and Amino Acid Metabolism

**Figure 12:**
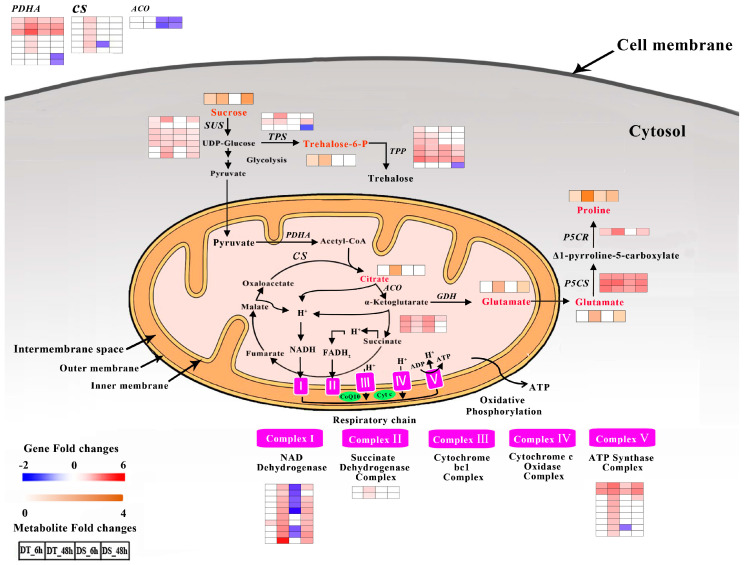
The pathways of carbon and amino acids metabolism. Genes (red and blue) and metabolites (brown and green) were displayed in different colors. The expression levels of genes are shown by a color gradient from low (blue) to high (red) in genes and from low (green) to high (brown) in metabolites. The squares are ordered from left to right: DT_6 h, DT_48 h, DS_6 h and DS_48 h. Numbers in the scale bar stand for the log_2_FC in expression.

In the correlation analysis of DEGs and DAMs, the carbon metabolism pathway and alanine, aspartate and glutamate metabolism pathways were specifically enriched in DT, while the arginine and proline metabolism pathway was enriched in both genotypes at 6 HAT. The DEGs are enriched in carbon metabolism, including starch and sucrose metabolism, glycolysis, pyruvate metabolism and the TCA cycle. The heatmaps in Figure 12 show expression for the DEGs and DAMs involved in these pathways. A total of 78 and 61 DEGs involved in these pathways were identified in DT and DS, respectively. All of these DEGs were upregulated in DT, while 17 DEGs were downregulated in DS, including one trehalose 6-phosphate phosphatase (*TPP*), one trehalose 6-phosphate synthase (*TPS*), two pyruvate dehydrogenase (*PDHA*), one citrate synthase (*CS*), four aconitate hydratase (*ACO*), seven Complex I (NADH dehydrogenase Complex) and one Complex V (ATP synthase) (Figure 12, Table S12). In addition, the overlapped upregulated DEGs showed higher expression levels in DT than DS, such as sucrose synthase (*SUS*), glutamate synthase (*GDH*), delta-1-pyrroline-5-carboxylate synthase (*P5CS*) and pyrroline-5-carboxylate reductase (*P5CR*).

A total of five DAMs were observed in these pathways under PEG treatment (Figure 12; Table S9). In the starch and sucrose metabolism, sucrose and trehalose 6-phosphate were significantly accumulated after PEG treatment in DT at both 6 and 48 HAT, while in DS, sucrose was significantly accumulated only at 48 HAT and trehalose 6-phosphate showed no changes under PEG treatment. The accumulation of citric acid in the TCA cycle was only observed in DT at 48 HAT in response to water stress. In the biosynthesis of the proline and glutamate pathway, proline and glutamate were much more significantly accumulated in DT than in DS after PEG treatment.

## 3. Discussion

### 3.1. Plant Growth

Plant roots play an essential role in water and nutrient uptake. Although the water contents of whole plant tissues decreased and cell elongation was inhibited under water stress [62], most plant species allocated more biomass to roots, increased root length and developed more and longer root hairs and lateral roots to promote water uptake [63]. In this study, DT showed higher dry weights (Figure 1a–c), water contents (Figure 1d,e) and longer RL, more RT, greater RA and RV than DS (Figure 2a–d). These results indicate that DT develops a higher size of root system that is more efficient for water and nutrient uptake compared to DS under PEG treatment and thereby shows higher drought tolerance to drought than DS.

### 3.2. DT Root Showed Higher ROS Scavenging Capacity under PEG Treatment

#### 3.2.1. Antioxidant Enzymes

The ROS mainly comprise H_2_O_2_, O_2_^•−^, ^1^O_2_ and OH^·^ and MDA is used as a marker of lipid peroxidation under oxidative stress [64,65]. Under abiotic stress conditions, plants use a scavenging enzyme system including SOD, CAT, POD, APX and GPX to hold ROS homeostasis and prevent oxidative stress [66]. GSTs can directly scavenge peroxides with glutathione as an electron acceptor and thus play a vital role in maintaining cellular redox homeostasis [67,68]. The overexpression of *Atgstu19* improves the tolerance to salt, drought and methyl viologen stresses in Arabidopsis [69]. In our study, we observed that a higher number of DEGs such as *SOD*, *POD*, *CAT*, *APX*, *GPX* and *GST* were upregulated and fewer DEGs including *POD* and *GST* were downregulated in DT than DS under PEG treatment (Figure 7a–f, Table S4). Furthermore, one ortholog *Atgstu19* was specifically upregulated in DT, indicating that the expression of GST genes was closely associated with drought resistance in wheat roots. The results of the physiological assay were consistent with the transcriptomic analysis, with higher activity of ROS scavenging enzymes observed in DT than DS (Figure 7g–l) under PEG treatment, as a consequence, the contents of root O_2_^•−^, H_2_O_2_ and MDA were constantly lower in DT than DS during the PEG treatment (Figure 3).

#### 3.2.2. Non-Enzymatic Antioxidants

As important non-enzymatic antioxidants, flavonoids protect plants from various biotic and abiotic stresses [8]. Anthocyanin, another non-enzymatic antioxidant, could not only remove free radicals but also prevent their production in plants under water-limited or salinity conditions [70]. The flavonoid biosynthetic pathway is orchestrated by a network of enzymes, including CHS, CHI, FLS, DRF and ANS [71]. UGT79B1 was recently identified to be involved in subsequent glycosylation of cyanidin 3-O-glucosides, which was the limiting step during anthocyanin synthesis in Arabidopsis [72]. Both anthocyanin and flavonoid were synthesized through the phenylpropanoid biosynthesis pathway, which was catalyzed by serial functions of PAL, 4CL and C4H [61]. In the phenylpropanoid biosynthesis pathway, PAL is a rate-limiting enzyme in response to stresses, such as drought, salinity and cold [61] and 4CL functions to convert *p*-coumarate into *p*-coumarate-CoA, a precursor of flavonoid [73]. In this study, most of these genes were upregulated in DT at two time points after PEG treatment. However, the genes including *DFR, F3′5′H* and *ANS* were almost downregulated in DS roots, especially at 48 HAT (Figure 11, Table S11). As a consequence, three anthocyanidin monomers (malvidin 3-O-glucosideand, cyanidin 3,5-O-diglucoside and delphinidin 3-O-glucoside) and four flavonoid metabolites (kaempferol 3-O-galactoside, quercetin, homoeriodictyol and hesperetin) were specifically accumulated in DT roots under PEG treatment relative to the DS roots (Figure 11, Table S9).

Transcription factor C1-MYB was reported to be an intersection of ABA-mediated signaling transduction in the anthocyanidin synthesis pathway [74]. In wheat, the transgenic lines with *ZmC1-MYB* transgene activated the expression of the anthocyanin biosynthesis-related genes including *TaCHS*, *TaCHI*, *TaF3H*, *TaF3′H* and *TaANS* [75]. In the present study, three orthologous *ZmC1-myb* were upregulated by water stress in both genotypes and showed higher expression levels in DT than DS, especially at 48 HAT (Figure 11, Table S3). The NCED enzyme regulates the key limiting step of ABA biosynthesis by catalyzing the cleavage of 9-cis-neoxanthin to xanthoxin [59]. The *osaao3* mutant exhibited decreased ABA levels, increased seedling growth while decreased drought tolerance compared to the wild-type [76]. In our study, a greater number of *NCED* genes were upregulated in DT (10) than in DS (4), while one gene in DT and three genes in DS showed downregulation. After PEG treatment, two genes annotated to be *AAO3* were upregulated in both genotypes and the expression levels of overlapped upregulated genes were much higher in DT than DS, in particular at 48 HAT (Figure 8b; Table S5). As expected, root ABA content was significantly increased in both genotypes after PEG treatment and was consistently higher in DT than DS (*p* < 0.05) (Figure 8e).

Considering the higher activity of ROS scavenging enzymes, higher accumulation of flavonoids and anthocyanin and lower ROS and MDA contents in DT than DS, we may conclude that DT had a higher ability to defend against oxidative stress under PEG treatment than DS. In addition, higher root ABA contents and activation of flavonoids and anthocyanin synthesis indicate that ABA signaling transduction might play an important role in drought-induced ROS scavenging in wheat roots.

### 3.3. AQPs

#### 3.3.1. Upregulation of Root AQP Expression Contributes to Water-Stress Tolerance

AQPs, a family of intrinsic plasma proteins, are closely associated with water transport [77]. The overexpression of *AQPs* in plants substantially improves root configuration and drought-stress tolerance [78]. AQPs are classified into five groups, such as PIPs, TIPs, NIPs, SIPs and XIPs [79,80]. The PIPs are classified into two subtypes, namely PIP1 and PIP2, based on their sequence. It is observed that PIP2s exhibit higher osmotic permeability, whereas PIP1s are inactive or display low activity [81]. TIPs regulate water transport in various vacuolar functions under drought or salt stress [82]. The overexpression of *OsPIP2;7* in transgenic plants enhances the ability of water uptake and rapid transport from roots to aerial tissues [83]. *OsPIP2;5*, encoding a root-specific aquaporin, contributed to the fine adjustment of radial water transport in roots to sustain high root hydraulic conductivity under water stress [84,85]. The expression of *OsTIP2;2* and *OsTIP4;1* contributes to the maintenance of water homeostasis and is significantly induced by dehydration, salinity and ABA treatments [82]. In this study, the expression levels of orthologous *OsPIP2;5* and *OsPIP2;7* were increased in both genotypes at 48 HATand DT showed more upregulated genes than DS. In addition, the genes encoding *OsTIP2;2* and *OsTIP4;1* were especially upregulated in DT while they showed no changes or were even downregulated in DS (Table S6).

#### 3.3.2. Hormones Play a Significant Role in the Regulation of AQP Expression in Response to Water Deficit

It had been reported that the AQPs’ gating regulation by phytohormones was involved in responses to environmental stresses by maintaining the water uptake and transport in plants [86,87]. Auxin promoted lateral root emergence by regulating the spatial and temporal distribution of aquaporin-dependent root tissue water transport at cell and whole-organ levels [88]. *TAA1* encodes a tryptophan aminotransferase that mediates the conversion of tryptophan to indole-3-pyruvate (IPyA) in the first step of IAA biosynthesis [89] and IPyA is subsequently converted to IAA via YUCCA enzymes [90,91]. In our study, a total of six DEGs including *TAA1* and *YUCCA* were upregulated in DT at both 6 and 48 HAT, whereas only three DEGs were upregulated in DS (Figure 8a; Table S5). As expected, the content of IAA was consistently higher in DT than in DS after PEG treatment (*p* < 0.05) (Figure 8d), which might contribute to the development of more lateral roots in DT than DS at 2 and 4 DAT (Figure 2d).

The accumulation of ABA in the root tips contributed to maintaining root elongation by its regulatory functions in osmotic adjustment at water deficit [92,93]. An increased level of ABA generally promoted a higher expression of root AQPs, which contributed to countering drought stress [77]. As discussed above, a greater number of *NCED* genes involved in the ABA biosynthesis were upregulated in DT (10) than in DS (6), the expression levels of overlapped upregulated *AAO3* genes were much higher in DT than in DS (Figure 8b, Table S5) and the root ABA contents were significantly and consistently higher in DT than DS at 6 and 24 HAT (*p* < 0.05) (Figure 8e). These results suggest that ABA may be involved in the modification of the root system and activate the expression of root AQPs to promote water absorption under PEG treatment (Figure 2).

The content of CK is generally reduced in drought stress [94] and thus promotes root system development, which is beneficial for plants grown on dry soils [95]. In poplar plants under mild drought conditions, the root with higher ABA and lower cytokinin tZR contents led to greater expression of the plant *AQP* genes (*PtPIP1;1*, *PtPIP1;2*, *PtPIP2;5* and *PtPIP2;7*) [96]. CKX is a key enzyme for the degradation of CK and enhanced activity of CKX altered the root system architecture, leading to increased root growth [97]. Our study found that more *CKX* genes were upregulated in DT than in DS and more *IPT* and *CYP735A* genes involved in CK synthesis were downregulated in DT than in DS (Figure 8c, Table S5). As a consequence, the root Z + ZR contents decreased significantly at 24 and 48 HAT in both genotypes (*p* < 0.05), with consistently lower values in DT than DS (*p* < 0.05) (Figure 8f).

In brief, phytohormone signaling may be involved in regulating root system architecture and the expression of AQPs and thereby promote efficient water absorption and transport during PEG treatment.

### 3.4. Lignin and Suberin Play Important Roles in Drought Tolerance

#### 3.4.1. Lignin and Suberin Deposition for Water Transport and Retention under PEG Treatment

Lignins are mainly composed of three hydroxycinnamyl alcohol monomers, including *p*-coumaryl alcohol, coniferyl alcoholand sinapyl alcohol, which produce *p*-hydroxylphenyl (H), guaiacyl (G) and syringyl (S) monolignol units, respectively [14]. Lignin deposition is important for maintaining water transport during and after drought by controlling the biomechanics of tracheary elements [98]. It is biosynthesized via a series of catalytic processes, in which PAL, C4H and 4CL were shared with the phenylpropanoid biosynthesis pathway and the *p*-coumaroyl CoA was channeled either further downstream into monolignol biosynthesis by CCR and HCT or into flavonoid biosynthesis by CHS [99]. *AtCCoAOMT* is induced under drought conditions and its mutation causes plants to become hypersensitive to drought [100]. *AtMYB61*, encoding a member of the R2R3-MYB family of TFs that regulates lignin biosynthesis as a transcription activator [101,102], is significantly upregulated under ABA treatment, which in turn significantly promotes the expression of key genes including *CCoAOMT, PAL*, *4CL* and *PRE* [103,104,105]. In the present study, the number of upregulated genes including *PAL, 4CL*, *CCoAOMT* and *POD* was greater in DT than in DS, especially at 48 HAT (Figure 11, Table S11). Of two *AtMyb61* genes, one was upregulated in both genotypes with a higher expression level in DT than DS at 48 HAT and another was specially downregulated in DS at 6 HAT. In the analysis of metabolic profiles, five DAMs (sinapyl alcohol, *p*-coumaryl alcohol, sinapic acid, caffeic acid O-glucoside and *p*-coumaraldehyde), classified as hydroxycinnamoyl derivatives, were especially enriched in DT, while they showed no significant changes and were even downregulated in DS (Figure 12, Table S9). Considering more upregulated and less downregulated DEGs involved in ABA biosynthesis (Figure 8b) and higher root ABA contents in DT than DS (Figure 8e), we suggest that ABA signaling transduction might play an important role in water transport by activating the genes involved in lignin biosynthesis pathway under water deficit.

Suberin deposition mainly occurs in the cell walls of the roots to form a barrier that separates living plant tissue from adverse environments and to prevent water and solute backflow from the roots to dry soil [106,107]. Therefore, the increased root suberization contributes to water retention under drought stress [18]. The suberin precursor is synthesized via enzymatic reactions of the KCS, FAR and CYP86 and is then transferred toward the cell wall by the ABCGs [108]. In Arabidopsis roots, compared to the wild type, the *atkcs2/daisy* mutant exhibited retarded growth and lower suberin composition [109]. Downregulation of *AtFAR1* and *AtFAR5* decreased suberin biosynthesis by reducing fatty alcohol contents in Arabidopsis roots [110]. The *cyp86a1 cyp86b1* mutant exhibited low suberin contents in periderm segments and showed increased water loss through the root periderm [111]. Compared to wild-type roots, the *atabcg1* mutant revealed reduced suberin content [60], while the *osabcg5* mutant had 50% less aliphatic suberin and their seed coats had seven-fold lower ω-hydroxyacid contents [112]. In this study, KEGG analysis showed that the suberin biosynthesis pathway was much more enriched in DT than DS (Figure 6) and the genes including *KCS*, *ABCG1* and *ABCG5* were all upregulated in DT, while one gene of *ABCG1* and three genes of *KCS* were specially downregulated in DS (Figure 9; Table S8). After PEG treatment, all of the *AtFAR1* and *AtFAR5* genes were upregulated in DT, while *AtFAR1* was specially downregulated in DS, in addition, DT showed more upregulated and higher expression levels of *CYP86A1* and *CYP86B1* genes than DS (Figure 9, Table S8).

Compared to DS, DT roots showed a much higher capability of water transport and water retention by promoting the synthesis of lignin and suberin under water deficit.

#### 3.4.2. Suberin and Lignin Regulate Lateral Root Formation via Phytohormone Signals

Lateral root is a key component of the root system architecture. During the lateral root initiation, the suberin overlying endodermal cells were first degraded; meanwhile, a lignin-based Casparian strip was also broken down to allow the growth of the lateral roots. Suberin was subsequently resynthesized in the endodermal cell walls, which contacted the lateral root primordium to support the emergence of the lateral root primordia [14,107]. GELPs, the lipid hydrolysis enzymes, played crucial roles in growth and development, stress responses and pathogen defense [113,114]. It had been reported that auxin-induced *GELP55* and *GELP72* were required for suberin degradation to facilitate lateral root emergence, while after prolonged auxin treatment, *GELP38* was repressed for the formation of lateral root primordia [107]. The ABA-induced expression of *MYB93* inhibited Casparian strip formation and promoted the expression of genes involved in multiple sub-networks in balancing lignin and suberin [115]. The double mutant *myb92-1 myb93-1* reduced suberin deposition in the root of Arabidopsis under water deficit [116]. In the present study, one *GELP55* gene was upregulated in DT while it was downregulated in DS and one *GELP72* gene was upregulated in both genotypes with a much higher expression level in DT compared to DS. Three *GELP38* genes were especially downregulated in DT while they were upregulated in DS at 48 HAT (Figure 9, Table S8). Furthermore, all of the genes including *MYB92* and *MYB93* were upregulated in DT at each time point, whereas they were specifically downregulated in DS at 48 HAT (Figure 9, Table S3).

In brief, considering the higher expression levels of upregulated DEGs included in the ABA and IAA biosynthesis pathway (Figure 8a,b, Table S5) and the higher contents of root ABA and IAA in DT than DS (Figure 8d,e), we suggest that DT has higher capability to develop more lateral roots by regulating the local degradation and deposition of lignin and suberin through ABA and IAA signaling compared with DS after PEG treatment. This could partly explain the higher tolerance to water stress in DT roots.

### 3.5. Production of Energy Sources for Root Growth and Development under PEG Treatment

As the direct source of cellular energy provision, ATP supports overall plant physiological activities, including growth and development, stress resistance, and crop quality [117,118]. It is produced through glycolysis and TCA cycle/oxidative phosphorylation under anaerobic and aerobic conditions, respectively [119]. Pyruvate dehydrogenase complex (PDC) is a mitochondrial matrix multienzyme complex that provides the link between glycolysis and the TCA cycle by catalyzing the conversion of pyruvate into acetyl-CoA [120]. Then, CS catalyzes the condensation of the acetyl moiety of acetyl-CoA with oxaloacetate to yield citrate [121,122]. Levi et al. (2011) observed that the accumulation of citric acid could contribute to a greater ability to resist drought stress in cotton [123]. In the TCA cycle, the oxidation of organic carbon substrates generates reducing equivalents (NADH and FADH_2_) that promote ATP synthesis by oxidative phosphorylation [124].

In our study, many more DEGs encoding PDHA, a member of PDC, were upregulated in DT (9) than in DS (6) at two-time points. All of the *CS* genes were upregulated in DT, while these genes showed no changes or were even downregulated in DS (Figure 12, Table S12). The number of upregulated DEGs encoding ATP synthase (Complex V) was much higher in DT compared to DS. In addition, the citric acid was observed only accumulated in DT. This indicated that DT had a higher capability to derive energy from the TCA cycle and oxidative phosphorylation for root growth and development under PEG treatment and might potentially explain the differences in water-deficit tolerance between the two genotypes in terms of biomass in our previous physiological studies.

### 3.6. Higher Osmotic Adjustment Improves Water-Stress Tolerance in DT

Proline, as an osmoprotectant, can not only maintain protein stability but also act as a metabolic signal of activation of an anti-stress response system, including ROS detoxification, induction of the specific gene expression, and protection of membrane integrity [125]. P5CS and P5CR are considered the key enzymes in proline synthesis. P5CS catalyzes the conversion of glutamate to Δ1-pyrroline-5-carboxylate (P5C), which is further reduced to proline by P5CR [126]. Soluble sugars, such as sucrose, trehalose-6-phosphate (T6P) and trehalose, were not only energy transporters but also essential hydrophilic solutes that protected cellular proteins and membranes from environmental challenges [127,128]. It had been reported that sucrose was accumulated in the roots of Arabidopsis [129,130], maize [131], rice [132] and pepper [133]. Trehalose, biosynthesized from T6P via biochemical reactions through TPS and TPP, was associated with tolerance and/or resistance of plants to drought [134,135].

In our study, the *P5CS* genes showed higher expression levels in DT than DS, especially at 48 HAT and *P5CR* was upregulated only in DT. The number of upregulated *TPS* and *TPP* genes was much higher in DT than in DS (Figure 12, Table S12). In addition, the accumulation levels of proline showed higher levels in DT than in DS, especially at 48 HAT (Figure 12, Table S9). The accumulation of sucrose and T6P in DT was increased at both 6 and 48 HAT, while in DS, sucrose was only increased at 48 HAT and no significant change was found in T6P. These findings were also consistent with our previous physiological research. In brief, compared with DS, DT showed a higher capability of maintaining cell osmotic pressure by producing a higher amount of osmoprotectants to defend against PEG-induced water stress.

## 4. Materials and Methods

### 4.1. Plant Growth and Treatments

Seeds of two common genotypes of wheat (drought tolerant; DT and drought-sensitive; DS) were cultivated as described by Hu et al. [58] with slight modifications. The 15-day-old seedlings were cultured in full-strength Hoagland’s nutrient solution [136] with 20% PEG 6000 to mimic drought stress or without PEG 6000 as controls.

### 4.2. Determination of Dry Weight and Water Contents

Seedlings were separated into shoots and roots to determine fresh weight (FW) at 2 and 4 days after treatment (DAT). The dry weight (DW) and water contents (WC) were defined as described by Hu et al. [58].

### 4.3. Root Measurements

The root architecture was determined using six plants, which were harvested at 0, 2 and 4 DAT and then the roots were scanned by using an EPSON1680 (WinRHIZO Pro2003b, Regent157 Instruments Inc., Quebec, QC, Canada) to measure RL, RV, RA, RT and RAD.

### 4.4. Determination of O_2_^•−^, H_2_O_2_ and MDA Content

The root samples were harvested at 6, 24, 48 and 72 h after treatment (HAT). The O_2_^•−^, H_2_O_2_ and MDA contents were determined by using kits (Suzhou Keming Biotechnology Co., Ltd., Suzhou, China). For determination of H_2_O_2_ and O_2_^•−^, samples (approximately 0.5 g) were grounded in 5 mL 100 mM pre-cooling phosphate buffer solution (PBS) and the homogenate was centrifuged at 12,000× *g*, 4 °C for 15 min, then the supernatant was used for measurement of H_2_O_2_ and O_2_^•−^ using the H_2_O_2_-1-Y kit and SA-1-G kit, respectively. For MDA, 0.5 g samples were grounded with 5.0 mL trichloroacetic acid solution (100 g/L) and centrifuged at 10,000× *g*, 4 °C for 15 min, the supernatant was used for measurement using an A003-1 kit. All treatments were repeated with three biological replicates.

### 4.5. Determination of Proline, Sucrose and Trehalose Contents

The fresh root samples (0.5 g) were harvested at 6, 24 and 48 HAT. Contents of Free proline, sucrose and trehalose were detected using their corresponding detection kits (Suzhou Keming Biotechnology Co., Ltd., Suzhou, China) at 520 nm, 480 nm and 620 nm, respectively.

### 4.6. Determination of Antioxidant Enzyme Activity

The roots were collected at 6, 24, 48 and 72 HAT and then frozen in liquid N_2_ and stored at −80 °C. The samples (0.5 g) were grounded into a fine powder under liquid N_2_. The powder was homogenized in 10 mL PBS (pH 7.8) and then centrifuged at 12000× *g* for 20 min at 4 °C. The supernatant was used to determine the activities of SOD, CAT, POD, APX, GPX and GST by using the SOD-1-W kit, CAT-2-Y kit, POD-1-Y kit, APX-1-W kit, GPX-A005-1 kit and GST-2-W kit (Suzhou Keming Biotechnology Co., Ltd., Suzhou, China), respectively.

### 4.7. Determination of Plant Hormones

The fresh root samples (0.5 g) were harvested at 6, 24 and 48 HAT and immediately preserved in liquid N_2_. The powders were extracted twice at 4 °C for 12 h with 1 mL pre-cooling 80% methanol. After centrifugation, supernatants were dried at 40 °C with N_2_. For the isolation of abscisic acid (ABA), indole acetic acid (IAA), zeatin (Z) and zeatin-riboside (ZR), the dried samples were redissolved in 0.5 mL methanol with 0.1 M glacial acetic acid were added for detection. In total, 100 μL was injected for HPLC analysis, the mobile phase was determined to be methanol/0.1 M acetic acid in a 4/6 (*v*/*v*) and the column temperature was 30 °C. The detection wavelength was 254 nm for ABA, GA, Z and ZR, while IAA was detected at an emission wavelength of 275 nm and an excitation wavelength of 345 nm.

### 4.8. RNA Extraction and Sequencing

Whole root tissues [total of 24 samples each with 10 plants] were collected, washed and frozen at −80 °C for the following RNA extraction. The total RNA was isolated as described by Hu et al. [58] and after confirmation of RNA integrity using Nanodrop™ and Experion™, the total RNA was sent to Exiqon™ for sequencing.

The RNA sample preparations utilized an input material of 3 μg RNA per sample. Sequencing libraries were generated as described by Hu et al. [58]. Briefly, the mRNA was purified from total RNA by using poly-T oligo-attached magnetic beads. To ensure the selection of cDNA fragments within the preferred length range of 150–200 bp, the library fragments were subjected to purification using the AMPure XP system (Beckman Coulter, Beverly, MA, USA) and quantification via Agilent Bioanalyzer 2100 system. Then, PCR was performed as described by Hu et al. [58]. After cluster generation, cDNA libraries were sequenced on the Illumina Hiseq™ 4000 platform to generate 150–200 bp paired-end reads (Metware Biotechnology Co., Ltd., Wuhan, China).

### 4.9. Transcriptome Sequencing Analysis

Raw data of the FASTQ format were first processed through in-house Perl scripts. The removal of adaptor sequences, unknown sequences ‘N’ and low-quality reads from raw data, was required for clean reads. By using the clean data, Q20, Q30, GC-content and sequence duplication were calculated. The clean reads mapped to wheat reference genomes on TopHat (http://tophat.cbcb.umd.edu/, accessed on 23 February 2016) were further analyzed and annotated.

Fragments Per Kilobase of transcript per Million mapped fragments Mapped (FPKM) were used to calculate and normalize the quantification of gene expression levels. A total of 12 selected genes were validated by qRT-PCR. The gene ID and primers are listed in Table S12. Differential expression analysis of two genotypes at two time points under normal and PEG conditions was performed with the Rbased package DESeq [137]. For controlling the false discovery rate (FDR), Benjamini and Hochberg’s approach was used to adjust the *p*-values. Genes with a FDR < 0.05 and a |log_2_FC(fold change)| ≥ 1 were defined as differentially expressed genes (DEGs).

### 4.10. The Gene Ontology (GO) and Kyoto Encyclopedia of Genes and Genomes (KEGG) Enrichment Analysis

Genes were annotated based on the Gene Ontology (GO) (http://www.geneontology.org, accessed on 8 September 2024) and Kyoto Encyclopedia of Genes and Genomes (KEGG) (http://www.kegg.jp, accessed on 1 September 2024) databases to obtain their functions. The significant KEGG pathway or GO enrichment of the genes was determined by selecting a Benjamini–Hochberg corrected *p*-value < 0.05. For each sample, the gene expression levels of three replicates were evaluated by Pearson’s correlation; the results are shown in a heatmap (Figure S3). Each R^2^ between three replicates was more than 95%.

### 4.11. Extraction for Widely Targeted Metabolic Profiling and High-Performance Liquid Chromatography (HPLC) Conditions

Chemical extraction was carried out on 24 samples (three biological replicates for both genotypes at two different time points under PEG treatment and control). Approximately 0.1 g of powder was extracted in 1.0 mL 70% aqueous methanol at 4 °C for 12 h. Following centrifugation at 10,000× *g* for 10 min, the extracts were absorbed and filtered prior to LC-MS analysis. The methods were previously described by Tian et al. [138].

The sample extracts were analyzed using an LC-ESI-MS/MS system (HPLC: Shim-pack UFLCSHIMADZU CBM30A system, www.shimadzu.com.cn/, SCIEX, Boston, Mass, USA; MS: Applied Biosystems 6500 Q TRAP). The analytical conditions were previously described by Lin et al. [139].

### 4.12. Widely Targeted Metabolic Profiling

A total of 24 samples were analyzed using a metabolomic platform that combined ultra-performance liquid chromatography (UPLC) and tandem mass spectrometry (MS/MS) [140]. The identification of metabolite was performed using the MWDB metware database (Metware Biotechnology Co., Ltd., Wuhan, China) and other public databases according to standard metabolic operating procedures.

### 4.13. Analysis of Metabolite Differences and Metabolic Pathways

The differential metabolites were screened by combining the fold change (FC) and the VIP value of the OPLS-DA model [141]. The difference in levels of selected metabolites between the control and the experimental group with |log_2_FC| ≥ 1 was considered to be significant and metabolites with VIPs ≥ 1 are considered to be significantly different. Different metabolic pathways were formed and constructed through the interactions between differential metabolisms in vivo. The differential metabolites were annotated and displayed using the KEGG database.

### 4.14. Correlation Analysis of Transcriptome and Metabolome

Correlation analysis was performed to examine the relationship between genes and metabolites detected in DT and DS at 6 and 48 HAT. Pearson’s correlation coefficients (PCC) among the DEGs and DAMs were calculated using the COR program in R language. DEGs and DAMs with a |PCC| > 0.8 were selected for further analysis. In this study, the DEGs and DAMs were also mapped to the KEGG pathway database to obtain their links in metabolic pathways.

### 4.15. Statistical Analysis

Data from three biological repeats were analyzed using SPSS software (v 20.0, Chicago, IL, USA) and rendered as the means ± SD. One-way ANOVA followed by Tukey’s significant deference test at *p* < 0.05 was utilized. All data had three biological repeats. Differences were considered statistically significant as described.

## 5. Conclusions

We conducted a comprehensive analysis of the global transcriptional and metabolic profiles and physiological determination in two wheat genotypes with contrasting drought tolerance. Our results suggest that the drought-tolerant genotype was less affected by water deficit at both the transcriptome and metabolome levels. Drought tolerance might be linked to (1) enhanced enzymatic and non-enzymatic antioxidants associated with ROS scavenging; (2) the improvement in the root system to take up water efficiently by regulating local lignin and suberin deposition and degradation via ABA and IAA signaling transductions; (3) enhanced water uptake and hydraulic conductivity by regulating the expression of AQPs through phytohormones; (4) high accumulation of osmoprotectants such as sucrose, proline and trehalose; and (5) the production of higher energy through the enhanced TCA cycle and oxidative phosphorylation.

## Data Availability

The data presented in this study are available in the Supplementary Materials. The reads produced in this study had been deposited in the National Center for Biotechnology Information (NCBI) SRA database with accession number SRP134221. Access to the data was available upon publication at http://www.ncbi.nlm.nih.gov/sra/, accessed on 31 March 2018.

## Supplementary Materials

The following supporting information can be downloaded at https://www.mdpi.com/article/10.3390/ijms251910430/s1.
