# Supplementary material for: Transcriptomic and Metabolomic Profiling of Root Tissue in Drought-Tolerant and Drought-Susceptible Wheat Genotypes in Response to Water Stress
Source: Int J Mol Sci. 2024 Sep 27;25(19):10430. doi: 10.3390/ijms251910430 (PMC11476764; doi:10.3390/ijms251910430)
Supplement: Supplementary file 1 [file ijms-25-10430-s001.zip › Fig. S1-S2.pdf]

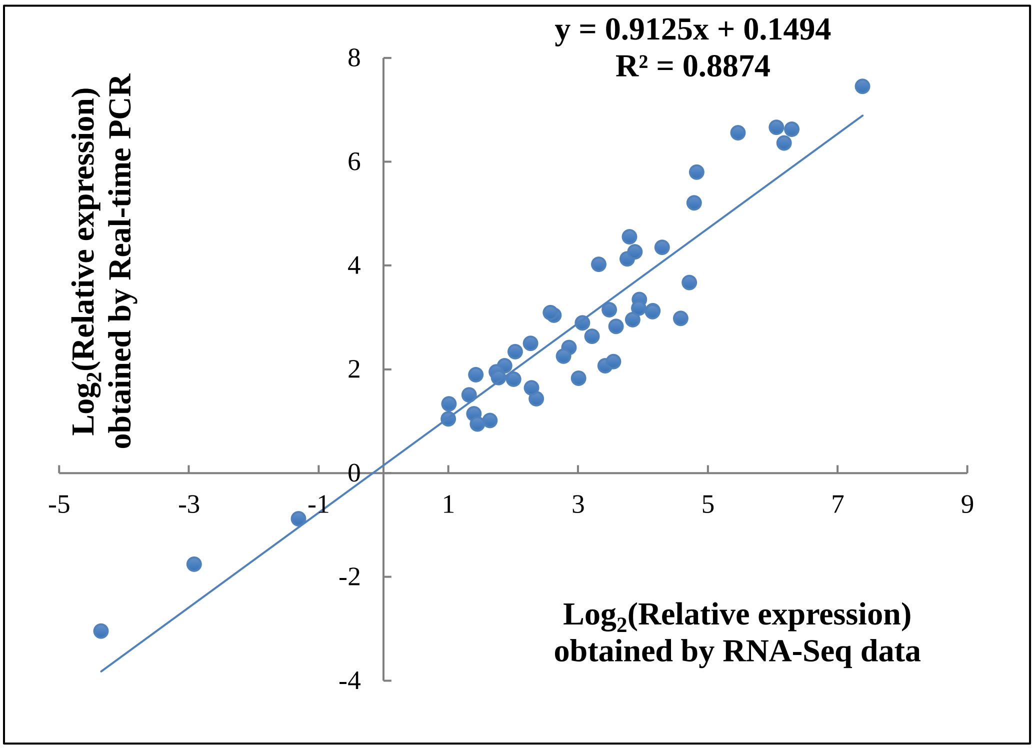

Fig. S1. 12 selected genes were validated by RT-qPCR method comparatively, and linear correlation between RNA-seq (x-axis) and RT-qPCR (y-axis) data was presented with a $\log_2$ (fold change) transformation. Primers are listed in (Table S12)

(a)

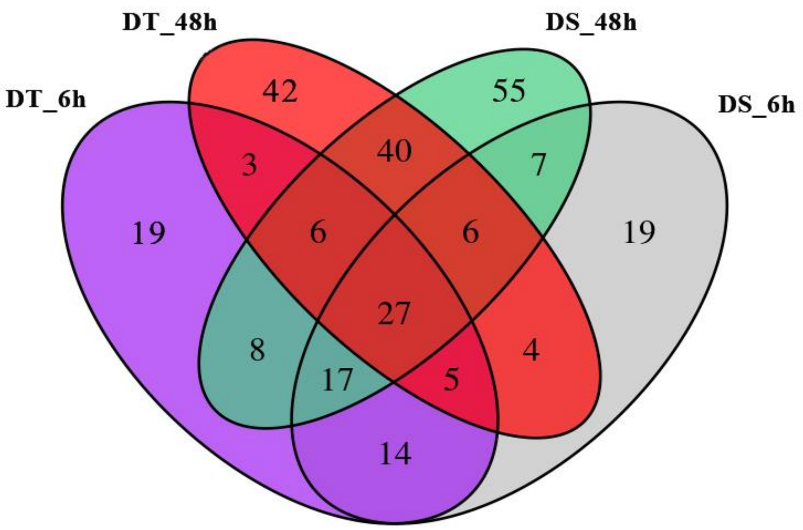

(b)

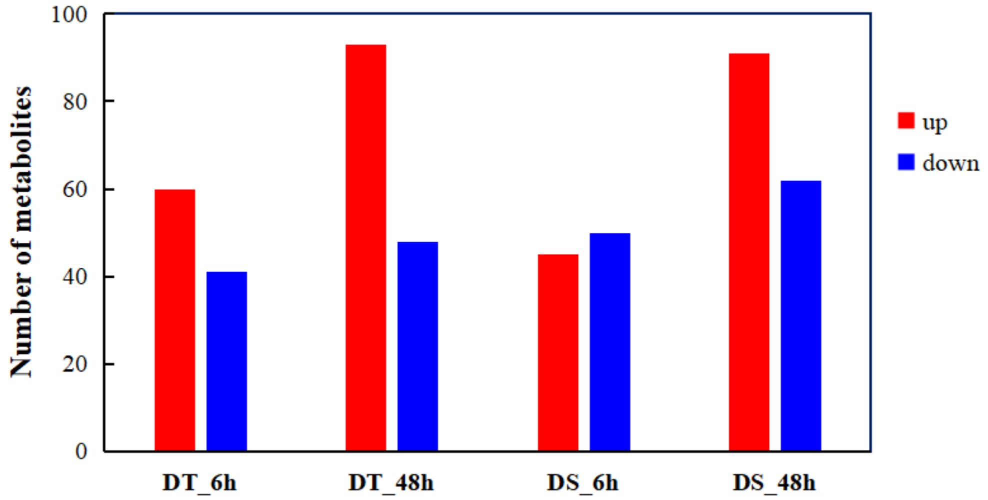

Figure S2. (a). Venn diagrams of DAMs in roots between DT and DS at 6 h and 48 h after PEG treatment. (b). The number of DAMs in roots between DT and DS at 6 h and 48 h after PEG treatment.
