# Supplementary figures and images for: Transcriptomic and Metabolomic Profiling of Root Tissue in Drought-Tolerant and Drought-Susceptible Wheat Genotypes in Response to Water Stress
Source: Int J Mol Sci. 2024 Sep 27;25(19):10430. doi: 10.3390/ijms251910430 (PMC11476764; doi:10.3390/ijms251910430)

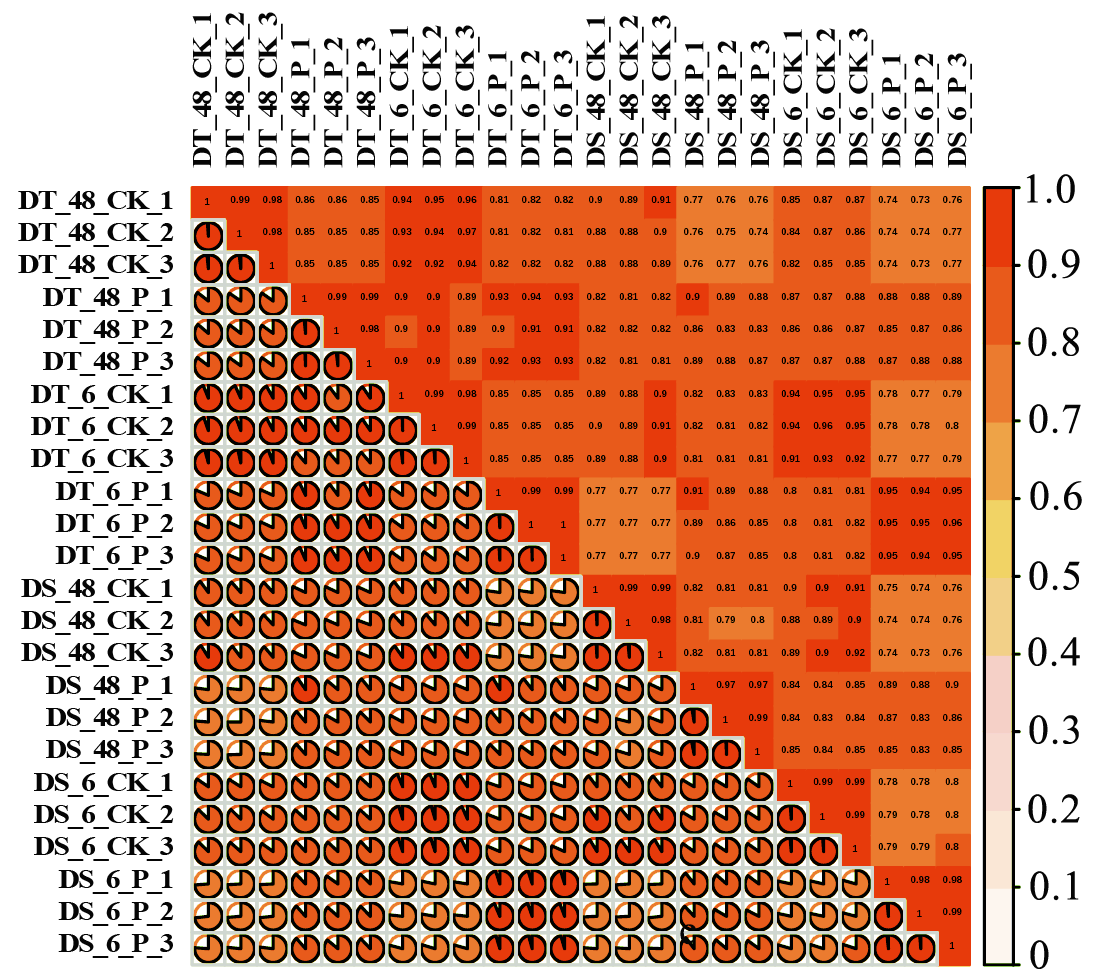

Supplement: Supplementary file 1 [file ijms-25-10430-s001.zip › Fig. S3.tif]
